# Direct comparison of ^68^Ga-DOTA-TOC and ^18^F-FDG PET/CT in the follow-up of patients with neuroendocrine tumour treated with the first full peptide receptor radionuclide therapy cycle

**DOI:** 10.1007/s00259-016-3328-2

**Published:** 2016-02-27

**Authors:** Bernhard Nilica, Dietmar Waitz, Vlado Stevanovic, Christian Uprimny, Dorota Kendler, Sabine Buxbaum, Boris Warwitz, Llanos Gerardo, Benjamin Henninger, Irene Virgolini, Margarida Rodrigues

**Affiliations:** Department of Nuclear Medicine, Innsbruck Medical University, Anichstrasse 35, A-6020 Innsbruck, Austria; Department of Radiology, Innsbruck Medical University, Anichstrasse 35, A-6020 Innsbruck, Austria

**Keywords:** ^68^Ga-DOTA-TOC, ^18^F-FDG, PET/CT, NET, PRRT

## Abstract

**Purpose:**

To determine the value of ^68^Ga-DOTA-TOC and ^18^F-FDG PET/CT for initial and follow-up evaluation of patients with neuroendocrine tumour (NET) treated with peptide receptor radionuclide therapy (PRRT).

**Methods:**

We evaluated 66 patients who had histologically proven NET and underwent both PRRT and three combined ^68^Ga-DOTA-TOC and ^18^F-FDG PET/CT studies. ^68^Ga-DOTA-TOC PET/CT was performed before PRRT, 3 months after completion of PRRT and after a further 6 – 9 months. ^18^F-FDG PET/CT was done within 2 months of ^68^Ga-DOTA-TOC PET/CT. Follow-up ranged from 11.8 to 80.0 months (mean 34.5 months).

**Results:**

All patients were ^68^Ga-DOTA-TOC PET-positive initially and at follow-up after the first full PRRT cycle. Overall, 62 of the 198 ^18^F-FDG PET studies (31 %) were true-positive in 38 of the 66 patients (58 %). Of the 66 patients, 28 (5 grade 1, 23 grade 2) were ^18^F-FDG-negative initially and during follow-up (group 1), 24 (5 grade 1, 13 grade 2, 6 grade 3) were ^18^F-FDG-positive initially and during follow-up (group 2), 9 patients (2 grade 1, 6 grade 2, 1 grade 3) were ^18^F-FDG-negative initially but ^18^F-FDG-positive during follow-up (group 3), and 5 patients (all grade 2) were ^18^F-FDG-positive initially but ^18^F-FDG-negative during follow-up (group 4).^18^F-FDG PET showed more and/or larger metastases than ^68^Ga-DOTA-TOC PET in five patients of group 2 and four patients of group 3, all with progressive disease. In three patients with progressive disease who died during follow-up tumour SUVmax increased by 41 – 82 % from the first to the last follow-up investigation.

**Conclusion:**

In NET patients, the presence of ^18^F-FDG-positive tumours correlates strongly with a higher risk of progression. Initially, patients with ^18^F-FDG-negative NET may show ^18^F-FDG-positive tumours during follow-up. Also patients with grade 1 and grade 2 NET may have ^18^F-FDG-positive tumours. Therefore, ^18^F-FDG PET/CT is a complementary tool to ^68^Ga-DOTA-TOC PET/CT with clinical relevance for molecular investigation.

## Introduction

Neuroendocrine tumours (NET) are a heterogeneous group of neoplasms characterized by their overexpression of somatostatin receptors (SSTR) on the cell surface [1]. Several studies have demonstrated the effectiveness of SSTR-targeted imaging for diagnosis, staging, follow-up and deciding upon the suitability of peptide receptor radionuclide therapy (PRRT) [2–5]. The standard indication for PRRT with the radiolabelled SSTR analogues ^177^Lu/^90^Y-labelled DOTA-Tyr3-octreotide (TOC)/TATE/lanreotide is metastatic and inoperable SSTR-positive NET, evaluated by SSTR imaging with ^68^Ga-DOTA-TOC/TATE/NOC/lanreotide PET/CT or ^99m^Tc-HYNIC-TOC/^111^In-DOTA-TOC/lanreotide scintigraphy, among others [6–10]. The former approach is preferable because of its superior resolution and hence better sensitivity than that of conventional scintigraphy. PET/CT imaging has also been shown to be superior to conventional imaging (CT, MRI) for staging [2, 3, 11].

NET typically have a wide range of cellular differentiation. WHO guidelines classify NET into three grades based on cell proliferation, the number of mitoses and the expression of the nuclear antigen Ki-67. Gastroenteropancreatic NET are classified as low grade (grade 1, Ki-67 <2 %), intermediate grade (grade 2, Ki-67 3 – 20 %) and high grade (grade 3, Ki-67 >20 %) [12]. Both proliferation index and grade strongly correlate with tumour behaviour and prognosis [10, 13, 14]. High-grade, poorly differentiated NET often have limited expression of SSTR [10], what can lead to false-negative SSTR imaging results and make the molecular investigation difficult.

^18^F-FDG PET/CT is used to assess glycolytic metabolism, and higher uptake of ^18^F-FDG has been found to be associated with tumour aggressiveness [15]. ^18^F-FDG PET/CT has thus been used increasingly in the recent years for the evaluation of high-grade NET [15, 16]. To date, however, only a few studies have investigated the correlation between ^18^F-FDG and SSTR imaging and NET grade. A dichotomous behaviour has been found between these approaches in well-differentiated and poorly differentiated NET, where the former was more positive on SSTR imaging [16, 17] and the latter on ^18^F-FDG PET [18–20]. Hence, adopting a dual-tracer approach encompassing SSTR and ^18^F-FDG PET imaging, assessing SSTR expression and glycolytic metabolism, respectively, could support better individualization of therapy selection in patients with NET. High ^18^F-FDG uptake would suggest an aggressive behaviour and the possibility of treatment refractoriness of the cells at the site, whereas low uptake would indicate a biologically indolent lesion.

We performed an intrapatient comparison of the results of ^68^Ga-DOTA-TOC and ^18^F-FDG PET/CT in the initial and follow-up evaluation of NET patients who had received the first full treatment cycle with PRRT. We also evaluated whether possible changes in tumour ^18^F-FDG uptake correlate with disease course.

## Materials and methods

### Patients

We retrospectively evaluated a cohort of 66 patients with histological confirmation of NET (according to the ENETS criteria) [13] who underwent PRRT (after confirmation of SSTR-positive lesions with ^68^Ga-DOTA-TOC PET/CT) and underwent three combined studies with ^68^Ga-DOTA-TOC and ^18^F-FDG PET/CT at our institution between 2005 and 2013. The methods of tissue collection were resection of the primary tumour in 27 patients, surgical excision of a metastatic lesion in 3 patients and biopsy of a metastatic lesion in the other 36 patients. The Ki-67 index was evaluated with immunohistochemistry. Patient demographics are shown in Table 1.

**Table 1:** Demographic data

Characteristic	Value
Total number of patients	66
Age at initial diagnosis (years)	
Mean ± standard deviation	57.2 ± 7.0
Range	34 – 78
Gender	
Male	38
Female	28
Primary tumour site	
Pancreas	20
Stomach	1
Jejunum	9
Ileum	15
Colon	2
Rectum	2
Lung	8
Unknown	9
Sites of metastases	
Liver	60
Lymph nodes	33
Bone	22
Lung	11
Adrenal gland	2
Brain	1
Thyroid gland	1
Spleen	1
Grade	
1	12
2	47
3	7

The data presented are number of patients, except age in years

All patients included in the study were in advanced stages requiring systemic antitumour therapy in a palliative setting. In particular, more than 65 % had metastases in more than one location, and most of them showed widespread metastases. Before undergoing PRRT, 43 patients were treated with other modalities including resection of the primary tumour (27 patients), chemotherapy (7 patients), and radiofrequency ablation or embolization of liver metastases (9 patients). The remaining 23 patients with widespread metastases were referred to our department for PRRT without previous therapy. Three patients were syndromic (two with insulinoma, one with gastrinoma). Fifteen patients had tumour progression at study entry. The average time from initial diagnosis to PRRT was 3.8 years (range 1 – 11 years, standard deviation ±1.4 years).

^68^Ga-DOTA-TOC PET/CT was performed at baseline (i.e. before PRRT), 3 months after completion of the first full PRRT cycle and every 6 – 9 months thereafter. All patients proceeding to PRRT underwent a ^18^F-FDG PET/CT scan as part of routine work-up. ^68^Ga-DOTA-TOC and ^18^F-FDG PET/CT were performed within 2 months of each other. Between these two scans no PRRT was given. A total of 198 combined ^68^Ga-DOTA-TOC and ^18^F-FDG PET/CT studies (baseline and after the first PRRT) were evaluated. Follow-up ranged from 11.8 to 80.0 months (mean 34.5 months).

### Peptide receptor radionuclide therapy

^90^Y-DOTA-TOC was used as the radiopharmaceutical of first choice for PRRT. ^177^Lu-DOTA-TATE was used if tumour lesions were smaller than 2 cm in diameter or in patients undergoing retreatment. ^90^Y-DOTA-TOC and ^177^Lu-DOTA-TATE were administered intravenously.

The periods between baseline evaluation and the first PRRT administration, and between RECIST 1.1 assessment and PRRT administration were <4 weeks. Three to four therapy cycles with 3.7 GBq ^90^Y-DOTA-TOC or 7.4 GBq ^177^Lu-DOTA-TATE were administered at an interval of 10 – 14 weeks. The treatment protocol used in our department [6] included the additional administration of “cold” long-acting SST analogues. These were administered after PRRT and repeated 4 weeks later. Retreatment with PRRT was performed at least 6 weeks after the use of “cold” long-acting SST analogues.

In 23 patients treatment was solely with ^90^Y-DOTA-TOC, in 22 patients solely with ^177^Lu-DOTA-TATE and in 21 patients with both agents sequentially. Thirty-five patients were retreated with PRRT. The number of therapy cycles with ^90^Y-DOTA-TOC ranged from 4 to 11 (cumulative activity 10.6 – 27 GBq) and with ^177^Lu-DOTA-TATE ranged from 4 to 9 (cumulative activity 16.3 – 37.5 GBq).

### Positron emission tomography

#### ^68^Ga-DOTA-TOC

Preparation of ^68^Ga-DOTA-TOC was based on a fully automated synthesis, as described previously [21]. The patients received 100 – 150 MBq of ^68^Ga-DOTA-TOC (20 – 30 μg) intravenously. The radiation exposure related to ^68^Ga-DOTA-TOC was 2.3 – 3.45 mSv [22]. PET acquisition was started 60 – 90 min (median 75 min) after injection. Imaging was performed with a dedicated PET scanner (MS-Advance or MS-Discovery 450; GE Healthcare). Images were acquired from the head to the mid-thigh. Attenuation correction was performed using transmission data obtained with a ^67^Ge pin source at 3 min per bed position (MS-Advance) or a CT scan (MS-Discovery 450). Ordered-subsets expectation maximization was used for image reconstruction.

#### ^18^F-FDG

Patients received 200 – 300 MBq of ^18^F-FDG intravenously after fasting for at least 8 hours. The radiation exposure related to ^18^F-FDG was 2.4 – 3.6 mSv [23]. PET acquisition was started 52 – 80 min (median 65 min) after injection. The settings and protocol were as described for ^68^Ga-DOTA-TOC.

#### CT

A 2.5-mm helical CT scan was performed on a HiSpeed CT/I Advantage scanner (GE Healthcare). Approximately 1.5 mL/kg body weight of Visipaque 320 contrast medium (GE Healthcare) was administered. The radiation exposure related to CT was 2 – 12 mSv [24].

### Image review

^68^Ga-DOTA-TOC and ^18^F-FDG PET images were assessed by two experienced board-certified nuclear medicine physicians. Criteria for a positive finding on PET studies were focal area(s) of increased tracer uptake or diffusely increased uptake, excluding physiological uptake, in comparison with adjacent tissue on axial, coronal and sagittal images. When the PET results corresponded with those of conventional imaging or histopathology, or when a corresponding lesion appeared on conventional imaging during follow-up, the PET results were rated as true-positive. Lesions not detected on PET but seen on conventional imaging and showing progression during follow-up or confirmed by histopathology were rated as false-negative. PET results suggestive of tumour lesions without corresponding lesions found on conventional imaging during follow-up or verification by histopathology were rated as false-positive.

All PET/CT images were analysed using commercially available software (eNTEGRA; GE Healthcare), which allowed review of PET, CT and fused imaging data. Semiquantitative analysis of all pathological lesions on ^18^F-FDG PET, calculating the maximum standardized uptake value (SUV_max_), was performed. For calculation of the SUV, regions of interest were drawn around areas with focally increased uptake on transaxial slices and automatically adapted to a 3-D volume of interest at a 70 % isocontour. The lesion with the highest SUV_max_ was chosen for data analysis. No SUV_max_ cut-off value was applied to differentiate benign from malignant lesions.

RECIST 1.1 was used for determining tumour response to treatment. Based on all imaging and histological findings as appropriated, tumour response was categorized as complete response (CR), partial remission (PR), stable disease (SD) or progressive disease (PD) [25, 26].

### Statistical analysis

SPSS software (version 18.0 for Windows SPSS Inc., Chicago, IL, and LEAD Technologies, Charlotte, NC) was used for statistical evaluation of the results. Continuous variables are expressed as mean values with standard deviations. The chi-squared test and *t* test were used. A *p* value <0.05 was considered statistically significant.

## Results

**Fig. 1:**
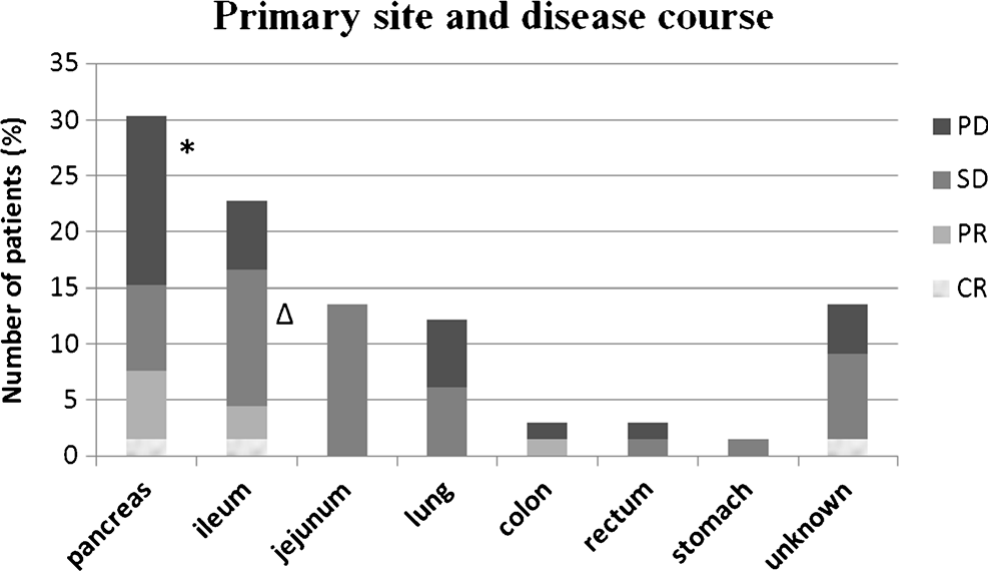
Disease course according to primary site (*CR* complete response, *PR* partial remission, *SD* stable disease, *PD* progressive disease) *p < 0.05 as compared with *SD* and *PR*; Δ *p* < 0.05 as compared with *PD*

**Fig. 2:**
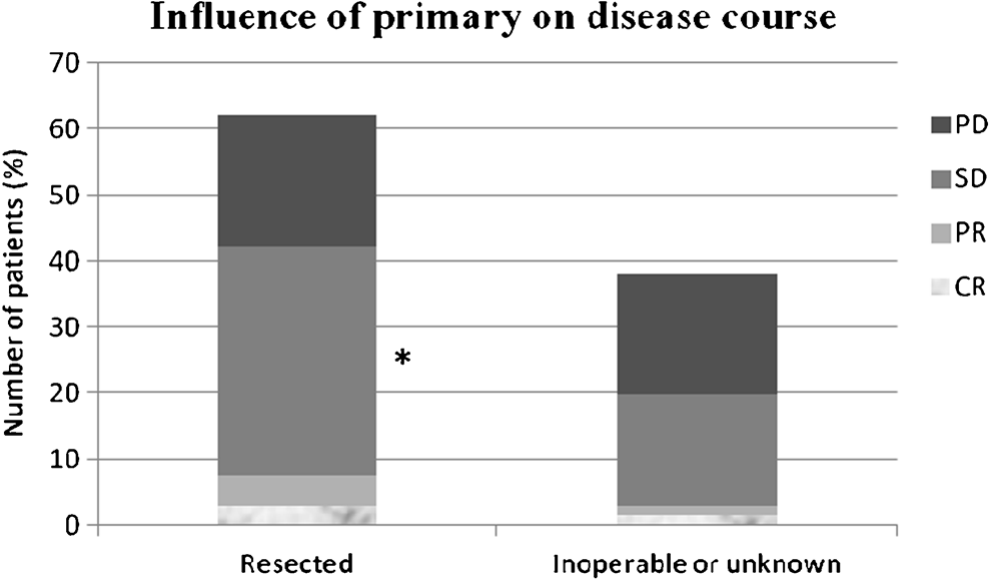
Influence of the primary tumour on disease course (*CR* complete response, *PR* partial remission, *SD* stable disease, *PD* progressive disease) *p < 0.05 as compared with group of inoperable or unknown primaries

The disease course at last follow-up was CR in 3 patients (4.5 %), PR in 4 (6.1 %), SD in 35 (53.0 %) and PD in 24 (36.4 %). Figure 1 shows the disease course according to primary site.Figure 2 shows the influence of the primary tumour on disease course.

### ^68^Ga-DOTA-TOC PET

All patients showed SSTR-positive tumour lesions at baseline and follow-up after the first full PRRT cycle. ^68^Ga-DOTA-TOC-positive lesions were primary tumours in 17 patients (25.8 %; pancreas in 12, lung in 4, ileum in 1 patient) and metastatic sites in 65 patients (98.5 %; liver in 63, lymph node in 32, bone in 23, lung in 8, brain in 1, adrenal gland in 1 patient).

### ^18^F-FDG PET

Overall, 62 of the 198 ^18^F-FDG PET/CT studies (31.3 %) were true-positive in 38 of the 66 patients (57.6 %). Two other patients showed ^18^F-FDG-positive lesions (lymph nodes in 2 patients, lung in 1 patient) only once during follow-up without correlating lesions on ^68^Ga-DOTA-TOC PET/CT and that were not seen on further follow-up investigations even though no therapeutic intervention took place. These two ^18^F-FDG PET/CT investigations were rated as false-positive.

### Analysis on a per-patient basis

**Fig. 3:**
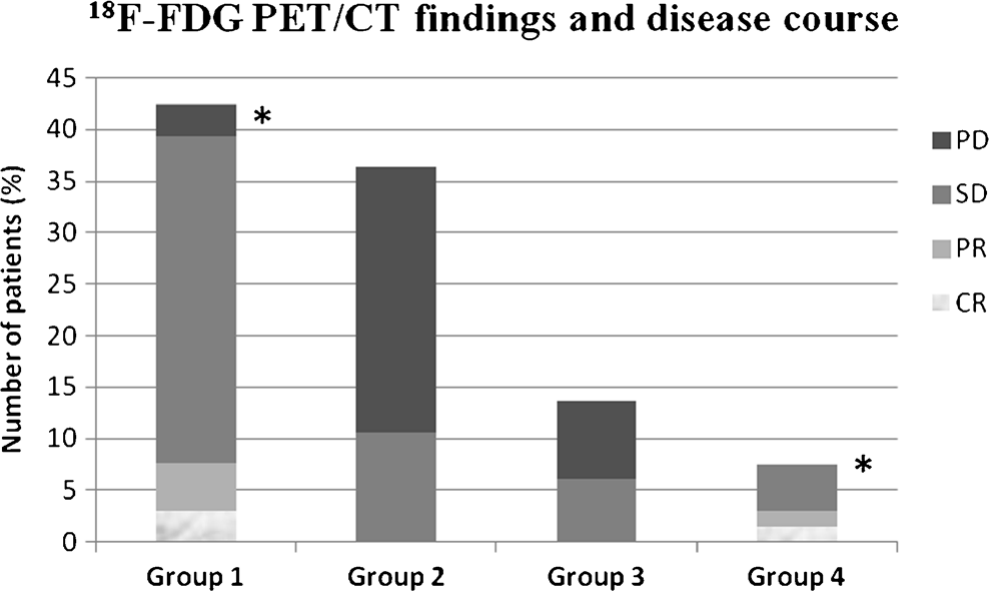
^18^F-FDG PET findings and disease course in each patient group: *Group 1* patients ^18^F-FDG-negative initially and during follow-up; *Group 2* patients ^18^F-FDG-positive initially and during follow-up; *Group 3* patients ^18^F-FDG-negative initially, but ^18^F-FDG-positive during follow-up; *Group 4* patients ^18^F-FDG-positive initially, but ^18^F-FDG-negative during follow-up (*CR* complete response, *PR* partial remission, *SD* stable disease, *PD* progressive disease; *p < 0.05 as compared with group 2

Patients were classified into four groups according to the ^18^F-FDG PET results as defined in the following sections. The ^18^F-FDG PET findings and disease course in each patient group are shown in Fig. 3.

#### Group 1

- Patients ^18^F-FDG-negative initially and during follow-up (28 patients, 42.4 %)

**Fig. 4:**
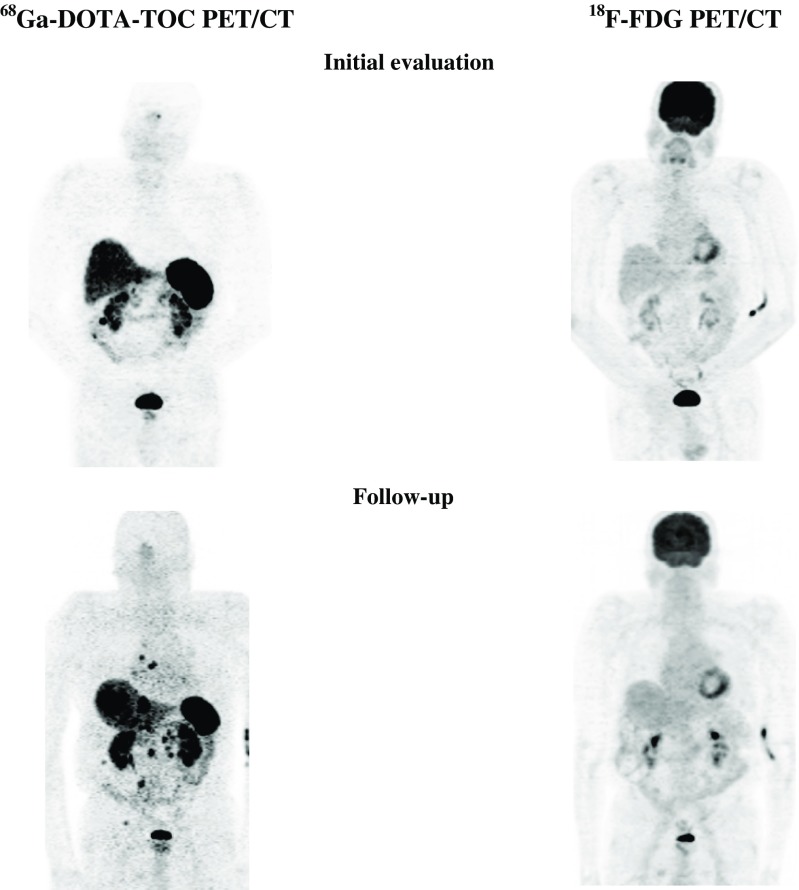
A 75-year-old male patient with grade 1 NET in the ileum. Positive ^68^Ga-DOTA-TOC PET (progressive disease) and negative ^18^F-FDG PET initially and during follow-up

^68^Ga-DOTA-TOC was positive in 2 primary tumours (2 patients) and in 52 metastatic sites (28 patients; Fig. 4).The tumour was grade 1 in 5 patients and grade 2 in 23 patients.

#### Group 2

- Patients ^18^F-FDG-positive initially and during follow-up (24 patients, 36.4 %)

**Fig. 5:**
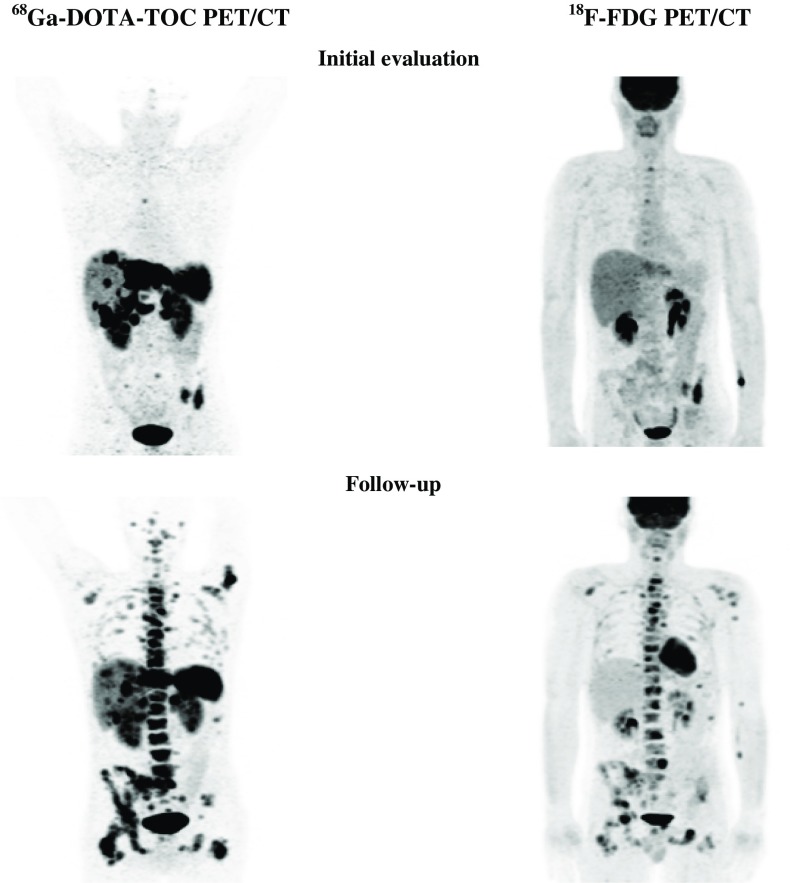
A 32-year-old male patient with grade 2 NET in the pancreatic tail. Positive ^68^Ga-DOTA-TOC PET and ^18^F-FDG PET initially and during follow-up (progressive disease)

^18^F-FDG PET was positive in 8 primary tumours (8 patients) and 44 metastatic sites (24 patients; Fig. 5). ^18^F-FDG PET showed more and/or larger metastases than ^68^Ga-DOTA-TOC PET in 5 patients (all with PD during follow-up). A ‘flip/flop’ phenomenon (with negative ^18^F-FDG PET but positive ^68^Ga-DOTA-TOC PET in some lesions, and positive ^18^F-FDG PET but negative ^68^Ga-DOTA-TOC PET in other lesions) was seen in 2 patients with PD.

^68^Ga-DOTA-TOC PET was positive in 9 primary tumours (9 patients) and 55 metastatic sites (24 patients). ^68^Ga-DOTA-TOC PET showed more metastases than ^18^F-FDG PET in 11 patients (3 patients with SD and 8 patients with PD during follow-up).

The tumour was grade 1 in 5 patients, grade 2 in 13 patients and grade 3 in 6 patients. Five patients died during follow-up (SD in 1 patient with grade 1, PD in 1 patient with grade 1, 2 patients with grade 2 and 1 patient with grade 3 tumour).

#### Group 3

- Patients ^18^F-FDG-negative initially but ^18^F-FDG-positive during follow-up (9 patients, 13.6 %)

**Fig. 6:**
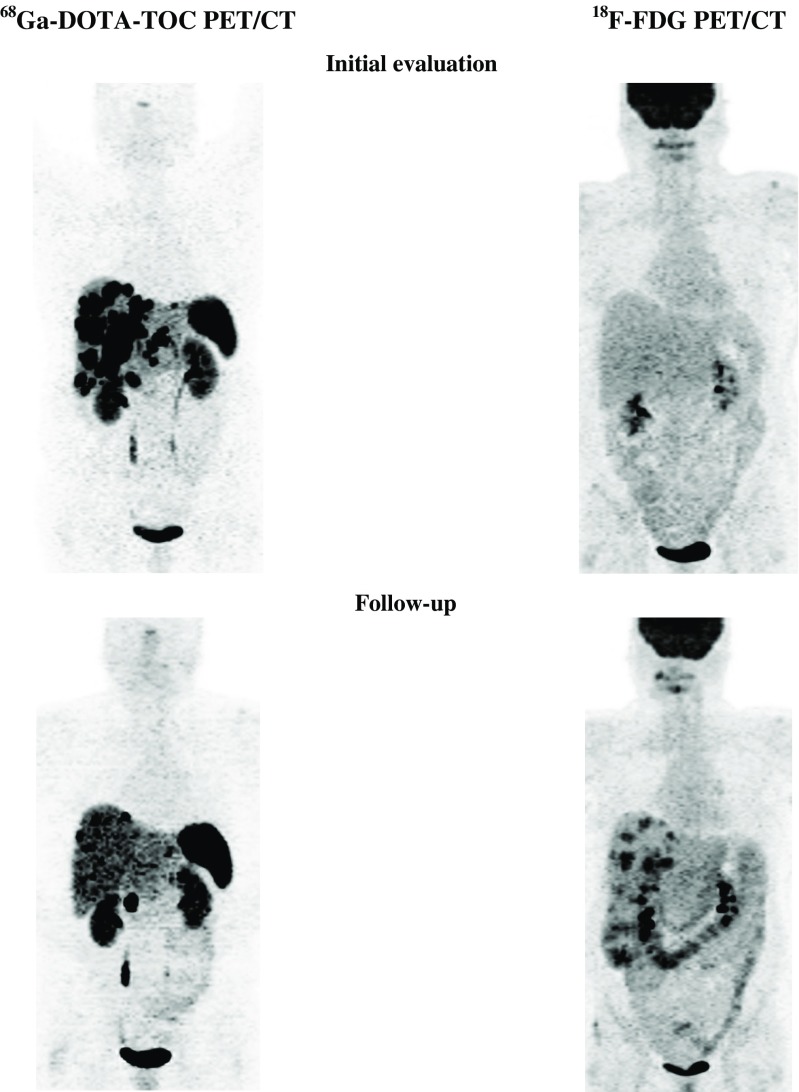
A 59-year-old female patient with grade 2 NET in the pancreatic head. Positive ^68^Ga-DOTA-TOC PET and negative ^18^F-FDG PET initially but positive ^18^F-FDG-PET/CT during follow-up (progressive disease)

^18^F-FDG PET was positive in 3 primary tumours (3 patients) and 12 metastatic sites (9 patients; Fig. 6). ^18^F-FDG PET showed more and/or larger metastases than ^68^Ga-DOTA-TOC PET in 4 patients (all with PD during follow-up).

Positive ^68^Ga-DOTA-TOC PET was seen in 3 primary tumours (three patients) and 13 metastatic sites (nine patients). The tumour was grade 1 in two patients, grade 2 in six patients and grade 3 in one patient.

#### Group 4

- Patients ^18^F-FDG-positive initially but ^18^F-FDG-negative during follow-up (five patients, 7.6 %)

**Fig. 7:**
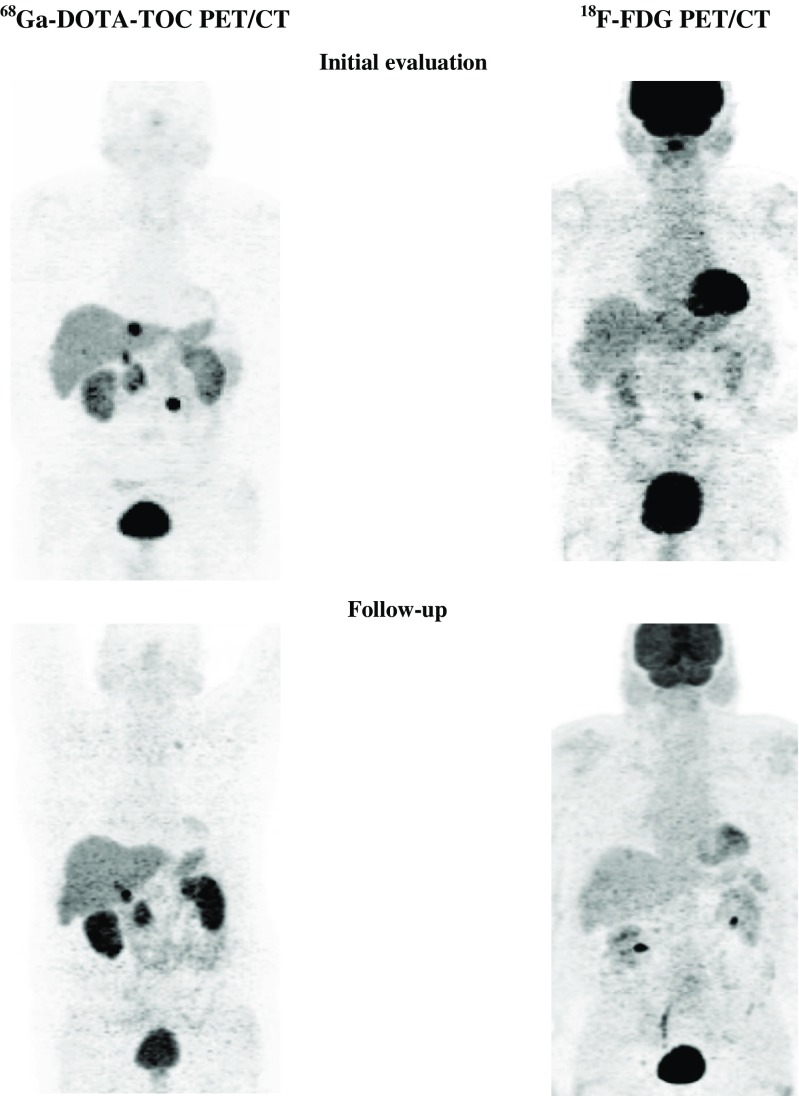
A 64-year-old male patient with grade 2 NET in the pancreatic head. Positive ^68^Ga-DOTA-TOC PET and ^18^F-FDG PET initially but negative ^18^F-FDG-PET during follow-up (partial remission)

Positive ^18^F-FDG PET was found in three primary tumours (three patients) and eight metastatic sites (five patients). Positive ^68^Ga-DOTA-TOC PET was seen in three primary tumours (three patients) and eight metastatic sites (five patients). The tumour was grade 2 in all patients. The disease course was CR in one patient (who was re-treated with PRRT and had radiofrequency ablation of liver metastases), PR in one patient (who was re-treated with PRRT; Fig. 7), and SD in three patients (one with surgical resection of primary tumour and LN metastases).

### SUV_max_ in ^18^F-FDG PET

In patients with ^18^F-FDG-positive lesions the SUV_max_ ranged from 3.0 to 13.0. No significant differences in SUV_max_ were found among the tumour grades or the various disease course groups. In patients with a more favourable disease course, the SUV_max_ in the primary tumour and metastases remained unchanged or showed a slight variation (5 % to 13 %) from first to last follow-up. However, in 3 patients with PD who died during follow-up, the SUV_max_ of the primary tumour (1 patient) and in various metastases (3 patients) showed an increase of 41 % to 82 % from first to last follow-up (SUV_max_ ranging from 11.2 to 11.9).

## Discussion

In the past few years, SSTR imaging with ^68^Ga-labelled peptide PET/CT has been shown to provide excellent sensitivity and specificity for diagnosing and staging NET [2, 3]. ^18^F-FDG PET is widely used in oncology, but its use in NET is a matter of controversy. Initial studies [20, 27], performed in a small number of patients only, cast doubt on the use of ^18^F-FDG PET in NET patients. The loss of SSTR expression was found to coincide with a gain in glucose utilization in tumours [28]. Consequently, it was suggested that the use of ^18^F-FDG PET be limited to SSTR-negative NET. More recent studies including a larger number of patients evaluated the xsensitivity of ^18^F-FDG PET in NET patients in comparison with that of ^68^Ga-DOTA-TATE PET/CT [16] and in relation to survival [29]. These studies showed that the higher the grade of NET, the higher is the prevalence of glucose hypermetabolic tumours [16], which have been linked with more aggressive tumour features including a higher risk of death [29]. This study confirmed that ^18^F-FDG positivity is strongly correlated with a higher risk of progression, in agreement with the findings of Garin et al. [30] showing that ^18^F-FDG PET has a prognostic value for early tumour progression. Furthermore, an important finding of our investigation is the evidence that patients may develop ^18^F-FDG-positive lesions during follow-up. This finding supports the value of repeating ^18^F-FDG PET in the long-term follow-up of NET patients, in particular if there are signs of progression on other imaging methods such as SSTR PET/CT.

Based on the Ki-67 proliferation index which is determined from analysis of a tumour sample taken mostly at the time of initial diagnosis, it is possible to stratify patients into different risk groups. However, a one-time collected parameter might not have the power to form the basis for therapeutic decisions at all stages and time-points during the disease course. Moreover, the collected tumour tissue might not be representative of all tumour lesions. For appropriate therapy planning it is important to detect the highest proliferative activity among all tumour lesions. SSTR imaging and ^18^F-FDG PET are modalities for noninvasive visualization of the whole body.

High ^18^F-FDG SUVs seem to be strongly correlated with short survival in NET patients. In accordance with Binderup et al. [29] who found that patients with a SUV_max_ higher than 9 are more likely to have a shorter overall survival, in 3 patients with progression who died during follow-up we found SUV_max_ in the range 11.2 to 11.9 in various tumours at the last follow-up, and importantly the values had increased 41 % to 82 % from the first follow-up. We did not perform a correlation study between ^18^F-FDG SUVs and Ki-67 index. Further prospective ongoing studies are necessary to establish the value of SUV in NET patients, in particular for assessing survival and progression-free survival.

The ENETS guidelines recommend that in patients with fast progression of NET classified as grade 1 or grade 2 a re-biopsy should be considered, but do not recommend ^18^F-FDG PET routinely during follow-up [31]. Guidelines now consider a place for ^18^F-FDG PET in a patient with grade 2 tumour in whom liver transplantation is planned to ascertain the absence of extrahepatic tumour lesions or other malignancies [10]. In this study, we confirm that patients with grade 1 or grade 2 NET may also have ^18^F-FDG-positive tumours initially and may develop ^18^F-FDG-positive lesions during follow-up. These findings must be taken into account, especially in individualized and optimized therapy planning.

The limitations of this retrospective study were the small number of patients in the subgroups, due in part to the heterogeneity of NET and the tumour primary sites, and the different treatments performed before and during follow-up. The treatment protocol used in our department included ^90^Y-DOTA-TOC as the radiopharmaceutical of first choice for PRRT. Although the possible superiority of ^90^Y for larger tumours has not been shown by direct comparison of ^90^Y-DOTA-TOC and ^177^Lu-DOTA-TATE, the rationale for using ^90^Y-DOTA-TOC in patients with tumours larger than 2 cm in diameter was based on data using a mathematical modelling approach based on the physical characteristics of therapeutic radionuclides [32]. A previous study has indicated that PRRT may lead to a reduction in glucose metabolism in NET lesions, depending on the amount of SSTR uptake as demonstrated by ^68^Ga-DOTA-NOC PET [9]. We evaluated mainly patients with grade 1 and grade 2 NET. Therefore, we were not able to further investigate the behaviour of grade 3 NET during the course of the disease with regard to ^18^F-FDG positivity of the tumour lesions.

In conclusion, our results show that investigation only of SSTR status by ^68^Ga-DOTA-TOC PET/CT may not reflect progression in certain NET lesions. Therefore, the decision on a change in therapeutic strategy in a patient with a poor prognosis cannot be based on this one modality alone. Hence, we recommend performing ^18^F-FDG PET in the initial evaluation and during follow-up of NET patients, especially when SSTR PET/CT shows progression. ^18^F-FDG PET/CT is a clinically relevant, complementary tool to ^68^Ga-DOTA-TOC PET and its use represents a step towards personalized medicine in the management of NET patients.
